# Effect of Aerobic Exercise on White Matter Tract Microstructure in Young and Middle-Aged Healthy Adults

**DOI:** 10.3389/fnhum.2021.681634

**Published:** 2021-07-02

**Authors:** David Predovan, Yunglin Gazes, Seonjoo Lee, Peipei Li, Richard P. Sloan, Yaakov Stern

**Affiliations:** ^1^Cognitive Neuroscience Division, The Taub Institute for Research on Aging and Alzheimer's Disease, Columbia University, New York, NY, United States; ^2^Department of Biostatistics, Columbia University, New York, NY, United States; ^3^Mental Health Data Science, New York State Psychiatric Institute, New York, NY, United States; ^4^Division of Behavioral Medicine, Department of Psychiatry, Columbia University, New York, NY, United States

**Keywords:** aerobic exercise, aging, cardiorespiratory fitness, cognition, white matter microstructure

## Abstract

Recent evidence suggests that being physically active can mitigate age-related white matter (WM) changes. In a randomized clinical trial, the effect of 6-month aerobic exercise (AE) or stretching/toning interventions on measures of WM microstructure (WMM) was assessed in a sample of 74 adults aged 20–67 years. Major WM pathways were reconstructed. No significant group-level change in WM tract microstructure following an AE training was observed. Without adjustment for multiple comparisons, an increase in fractional anisotropy (FA) and a decrease in mean diffusivity (MD) of the uncinate fasciculus were observed post-intervention in the AE group in comparison with the stretching group. In the AE group, a significant increase in cardiorespiratory fitness was measured but did not correlate with FA and MD change. The present results of this study are in accordance with similar studies in healthy adults that did not show significant benefit on WMM after participating in an AE program.

**Clinical Trial Registration:** Clinicaltrials.gov identifier, NCT01179958.

## Introduction

Intervention-induced white matter (WM) plasticity has been seen using animal models (for example, the effect of motor skills learning in rats; Sampaio-Baptista et al., 2013) and in humans (training of a complex visuo-motor skill; Scholz et al., 2009). For a review of the literature on WM plasticity, see Sampaio-Baptista and Johansen-Berg (2017). In older adults, some evidence suggests that a higher level of physical activity (Gow et al., 2012; Sexton et al., 2016; Strommer et al., 2020) and high cardiorespiratory fitness (CRF) (Johnson et al., 2012; Oberlin et al., 2016) may attenuate age-related alterations in the WM microstructure (WMM) (Burzynska et al., 2010; Cox et al., 2016; Damoiseaux, 2017; Slater et al., 2019; Wassenaar et al., 2019). The association between CRF and WMM might be age-dependent, as CRF was positively associated with WMM in older adults (age 55–82), but not in younger adults (age 18–31) (Hayes et al., 2015). In older adults, Colcombe et al. (2003) reported a significant beneficial effect of CRF on WM anterior tracts and in transverse tracts running between the frontal and posterior parietal lobes. Furthermore, in older individuals, an increase in WM volume in prefrontal and temporal cortices was reported after participating in an aerobic exercise (AE) training (Colcombe et al., 2006). Also in older adults, Burzynska et al. (2017) reported an increase in the integrity of the fornix, based on fractional anisotropy (FA), upon completion of 6-month dance programs, compared with a decline in the other interventions (walking, walking + nutrition, and stretching and toning). Voss et al. (2013), investigating the effect of a 12-month AE program (walking) in older adults, reported no significant change in WMM, although a positive correlation was observed between training-induced CRF related to the AE program and prefrontal and temporal FA.

There is a noticeable gap in the literature assessing the benefit of physical exercise training on the brain and cognition due to the dearth of studies on young to middle-aged adults (Erickson et al., 2019; Stillman et al., 2020). Examining this understudied population allows us to assess if it is possible to mitigate age-related declines more proactively at an earlier age. This avenue of research could have important public health implications. In middle-aged adults, considered 40–65 years (Irwin et al., 2018), higher CRF was associated with higher FA measured 5 years later (Zhu et al., 2015). In this population, little is known about the effect of early intervention promoting physical activity and CRF, particularly whether it is prone to mitigate age-related alterations in the WMM. Little is also known on sex differences in exercise efficacy to improve cognition (Barha et al., 2017) and brain integrity in this age group. In this randomized clinical trial (RCT) (Stern et al., 2019a), potential sex moderation was found on exercise-related changes in executive function (EF), such that in any age range, men (in the AE group) improved more on EF than women, but it remained to be seen if improvement in WMM integrity could be the underlying cause of this change.

The objective of the present study was to fill that void using previously collected data, including diffusion tensor imaging (DTI) data such as FA and mean diffusivity (MD), from a completed RCT (NCT01179958 on Clinicaltrials.gov). In this RCT (Stern et al., 2019a,b), 132 healthy adults aged 20–67 with below-median CRF [assessed by graded exercise test (GXT)] were randomly assigned to either a supervised 6-month AE program (known to increase CRF, cognitive performance assessed by a neuropsychological assessment battery, and brain health in healthy older adults; Gaertner et al., 2018; Northey et al., 2018; Stimpson et al., 2018; Falck et al., 2019) or a stretching/toning program. It was hypothesized that (1) in comparison with the stretching group, the AE group will show a significant increase in FA and a significant decrease in MD in the fornix and in the WM tracts related to frontal regions. (2) This effect will be modulated by age, such that older participants will obtain greater benefit. (3) In the AE group, a significant positive correlation will be found between intervention-related WMM and change in cognitive performance, particularly EF, as a significant association between EF and FA was reported using regional and voxel-based analyses (Grieve et al., 2007) and tractography analysis (Li et al., 2018). (4) In the AE group, a significant positive correlation will be found between intervention-related WMM and CRF change. 5) Lastly, we will ascertain if the improvement of WMM integrity is a possible mechanism by which men obtained greater cognitive benefits than women as was found in this RCT in which sex moderated the effect of the AE training on cognitive benefit (Stern et al., 2019a), and thus, sex was included as a covariate for all analyses in this study.

## Materials and Methods

### Participants

Participants were recruited in New York City through posted flyers and social media. Screening of the inclusion/exclusion criteria was done through a phone interview and subsequently during in-person visits. To be included in the RCT, participants had to be healthy, cognitively intact (a score > 135 on the Mattis Dementia Rating Scale), right-handed (based on the Edinburgh Handedness Questionnaire), non-smoking, sedentary, and habitual non-exercisers (a score < 2 on the Baecke Physical Activity Sports). Furthermore, participants were required to have below-average CRF (as categorized by the American College of Sports Medicine and the American Heart Association standards) determined by a GXT on an electronic-braked cycle ergometer. The study protocol and informed consent form were approved by the Institutional Review Board of New York State Psychiatric Institute. The trial was overseen by an independent data and safety monitoring board under the auspices of the National Institute on Aging. All participants provided written informed consent before enrollment. The present study only includes a subset of the RCT participants who had diffusion-weighted imaging (DWI) data. At baseline, 74 participants had DWI data, of which 73 had longitudinal TRActs Constrained by UnderLying Anatomy (TRACULA) data.

### Procedure

Recruitment and physical training programs were conducted from May 2011 to April 2016. Prior to randomized assignment to either the AE or the stretching intervention condition, participants eligible for the RCT were enrolled in a run-in period. The run-in period took place in five YMCA's (New York City fitness centers) in Manhattan, lasted 2 weeks (three sessions/week), and did not incorporate AE training. To continue in the intervention phase of the study, participants had to attend at least five sessions during the run-in period. Eligible trial participants were randomly assigned to the two conditions with an allocation ratio of 1:1. The randomization schedules were generated by the study statistician and concealed until an eligible participant was ready for enrollment. The AE or the stretching/toning program was 6 months in length and consisted of four structured 60-min exercise sessions per week. Participants went to the fitness center according to a schedule they determined. Fitness center trainers introduced the exercise programs to the participants. All training sessions in both conditions consisted of 10–15 min of warm-up/cool down and 30–40 min of workout. Participants were contacted on a weekly basis by coaches to monitor their progress. In the AE program, participants were selected from a series of aerobic activities (treadmill, elliptical trainer, and stair stepper). Participants were instructed to exercise alone. Throughout the exercise sessions, participants wore a Polar Electro model s610i to assess their heart rate (HR). Participants were instructed to train at a specific percentage of their maximal HR (weeks 1–2, 55–65%; weeks 3–4, 65–75%; weeks 5–24, 80%). Data from each participant were downloaded into a computer located in each fitness center. The maximal HR was determined at baseline by a GXT. The stretching/toning program consists of a series of exercises designed to promote flexibility and improve core strength. Core strengthening exercises included the back, abdomen, and pelvic muscles. Participants were told to hold each stretch for 15–30 s. Each stretch was repeated 10 times. Neuropsychological assessment and CRF assessment were completed prior to randomization (baseline), at the midpoint (month 3), and upon completion of the intervention (month 6). MRI was acquired at baseline and month 6. For a detailed description of the comprehensive neuropsychological assessment, CRF assessment, and processes related to the MRI data acquisition and preprocessing, readers are referred to the original study by Stern et al. (2019b).

### Neuropsychological Assessment

Cognition was divided into six domains: executive function [the local switch costs from a Set switching task and the total rule break errors across five trials of the Groton Maze Learning Test (CogState; Lim et al., 2013)], episodic memory [the delayed recall score from the Modified Rey Auditory Verbal Learning Test and the number of errors from the Continuous paired associate learning test (CogState)], processing speed [the number of symbols copied from the Wechsler Adult Intelligence Scale—Third Edition (WAIS-III) digit symbol subtest and the number of clicks per second from the Groton Maze Chase Test (CogState)], and the reaction time from the Identification task (CogState), language (the total words produced across three letters on the Controlled Oral Word Association Test and the number of animal names produced in 60 s on the Animal naming test), attention (the total number of correct cancellations within 5 min on the two and seven tests), and working memory [the total number of correct sequence trials on the WAIS-III letter–number sequencing and the correct responses on the N-back task (CogState)]. Each domain is represented by a summary *z* score. The specific measures used for each cognitive domain outcome were selected prior to initiating analyses. The selection of tests for each cognitive domain outcome was validated using factor analysis of the baseline test data, which produced groupings comparable with those included in the listed cognitive outcomes. Standardization of each test was based on mean and *SD* of baseline values. Mean values of the standardized outcomes of the tests in each cognitive domain were used for analysis.

### Cardiorespiratory Fitness Assessment

Participants' CRF was indexed by an estimate of their maximal oxygen uptake (VO_2_ max) obtained on a GXT with an electronic-braked cycle ergometer (Lode Corival, Groningen, the Netherlands), which was connected to a metabolic measurement cart (Ultima CPXTM; MedGraphics, St. Paul, MN, USA). A ramping protocol (10, 15, or 20 W, 2 min each) was personalized to each participant's perceived exercise capacity to yield a test duration of approximately 10 min. After a 2-min warm-up against no resistance, the work rate increased linearly at the individualized ramp rate until volitional fatigue. ECG and respiration gas exchange variables (VO_2_, VCO_2_, expired ventilation, and respiratory exchange ratio) were continuously monitored and recorded during the exercise test. Every 2 min, blood pressure and perceived exertion (Modified Borg 0–10 scale) were also measured. The highest 15-breath moving median for the test was considered VO_2_ max. Verification criteria for the determination of VO_2_ max were as follows: a respiratory exchange ratio >1.1, a plateau in the slope of the VO_2_-work rate relationship, and an HR over 90% of the maximum age predicted HR limit. The equation HRmax = 220—age was used to compute the maximum age-predicted HR limit (Fox et al., 1971).

### MRI Data Acquisition

MR images were collected with a 3.0T Philips (Best, the Netherlands) Achieva MRI scanner. For each participant, a T1-weighted magnetization-prepared rapid gradient echo (MPRAGE) scan of the whole brain was acquired, with echo time/repetition time (TE/TR) of 3/6.5 ms and flip angle of 8°, in-plane resolution of 256 Å ~ 256, field of view of 25.4 Å ~ 25.4 cm, and 165~180 slices in axial direction with slice thickness/gap of 1/0 mm. A neuroradiologist evaluated the T1 scans for clinically significant findings. Two sets of DWI scans were acquired in 56 directions using the following parameters: b = 800 s/mm^2^, TE = 69 ms, TR = 11,032 ms, flip angle = 90°, in-plane resolution 112 Å ~ 112 voxels, acquisition time = 12 min 56 s, slice thickness = 2 mm (no gap), and 75 slices.

### T1-Weighted Structural Image Parcellation

In order to extract major WM tracts using TRACULA, an automated tractography tool distributed as part of the FreeSurfer 6.0 library (Yendiki et al., 2011, 2016), the longitudinal pipeline in FreeSurfer 5.1 was used to first parcellate the T1-weighted image into gray matter and WM and cerebrospinal fluid. In the longitudinal processing stream, an unbiased within-subject template space and image (Reuter and Fischl, 2011) was created using robust, inverse consistent registration (Reuter et al., 2010) within each subject. Several processing steps, such as skull stripping, Talairach transforms, atlas registration, and spherical surface maps and parcellations are then initialized with common information from the within-subject template, significantly increasing reliability and statistical power (Reuter et al., 2012). Gray matter regions were then parcellated into 34 regions per hemisphere using the Desikan–Killiany Atlas (Fischl et al., 2004). These regions served as anatomical landmarks during tractography, which maximized the accuracy of the tracts to be extracted. These parcellations were manually checked, and if errors were found, controls points were adjusted and the parcellation was rerun, thus ensuring the accuracy of the final parcellations. T1-weighted image from each time point was processed separately.

### Diffusion Tensor Imaging Data Processing

For each time point, the two DWI scans were concatenated and similarly for the b-vector and b-value files. The merged DWI data for both time points were processed using the longitudinal stream in TRACULA (Yendiki et al., 2016). The longitudinal stream of TRACULA used the within-subject template from the FreeSurfer parcellation step for the anatomical space within which to conduct tractography. Standard DWI preprocessing steps were first conducted using tools from the FMRIB's Diffusion Toolbox (FMRIB's Software Library v. 5), including eddy current and movement corrections (Andersson and Sotiropoulos, 2016). All rotations of the volumes in the preprocessing steps were also applied to the vector directions. Upon preprocessing, DTI models were estimated to produce FA and MD measures at each voxel. To enable probabilistic fiber tractography, FSL's BedpostX was also performed and subsequently used by TRACULA to extract 18 major WM tracts: bilateral corticospinal tract (CST), bilateral inferior longitudinal fasciculus (ILF), bilateral uncinate fasciculus (UNC), bilateral anterior thalamic radiation (ATR), bilateral cingulum–cingulate gyrus (supracallosal) bundle (CCG), bilateral cingulum–angular (infracallosal) bundle (CAB), bilateral superior longitudinal fasciculus–parietal bundle (SLFP), bilateral superior longitudinal fasciculus–temporal bundle (SLFT), forceps major (FMAJ) (which passes through the splenium of the corpus callosum), and forceps minor (FMIN) (which passes through genu of the corpus callosum). The FA and MD were then extracted for each pathway at each time point.

As TRACULA does not provide tract reconstruction of the fornix, 25% probability masks from the Johns Hopkins University WM labels atlas were used to extract FA and MD for the fornix (Gazes et al., 2018). To ensure robust estimation of longitudinal changes in FA and MD, the base template from FreeSurfer T1 processing was also used as the template space from which to extract the FA and MD for each time point. In all analyses, bilateral tracts were averaged to generate one mean value per tract. For each participant, the FA, a summary measure of microstructural integrity, and MD, an inverse measure of the membrane density (Alexander et al., 2011), were computed.

Since there is no official guideline on quality checks for TRACULA output, we adopted stringent inclusion criteria for tracts generated from TRACULA in order to ensure only properly generated tracts were included in the analysis: each of the 18 WM tracts for each participant at each time point was visually inspected in FreeView with the default settings. Any tract that did not display as a full tract was reprocessed through TRACULA with control points being modified. This was attempted twice for each problematic tract. Any tract that was not recoverable with reprocessing was excluded from the analysis. No scans were excluded on the basis of poor scan quality.

### Statistical Analysis

Baseline demographic characteristics, CRF, and cognitive domains were compared between the intervention groups using Wilcoxon's rank test and Fisher's exact test for continuous and categorical variables, respectively.

Mixed ANOVA was used to test the effects of the intervention on cognitive performance and CRF outcomes. Two mixed ANOVAs (one for each WMM) were conducted to compare the intervention groups on the respective diffusion metrics (FA and MD). The role of age in intervention-related changes to the WM tract microstructure was investigated by evaluating a three-way interaction (Time × Age group × Intervention group). Age group was determined by the median age of the whole sample (39 years) and then splitting the sample into two age groups: young adults [age 20–39, mean age 29.19 years (*n* = 33)] and middle-aged adults [age 40–67, mean age 55.30 years (*n* = 37)].

*Post-hoc* correlations were conducted to assess the relation between intervention-related percentage change in terms of WMM, CRF, and cognitive performance. In all analyses, the reported *p*-values are two-tailed, and the significance level was set to 0.05. Multiple comparison correction controlling for false discovery rate (Benjamini and Hochberg, 1995) was performed for the WM tracts. All analyses were done using IBM® SPSS® Statistics Version 25.

## Results

### Participant Baseline Comparability

**Figure 1:**
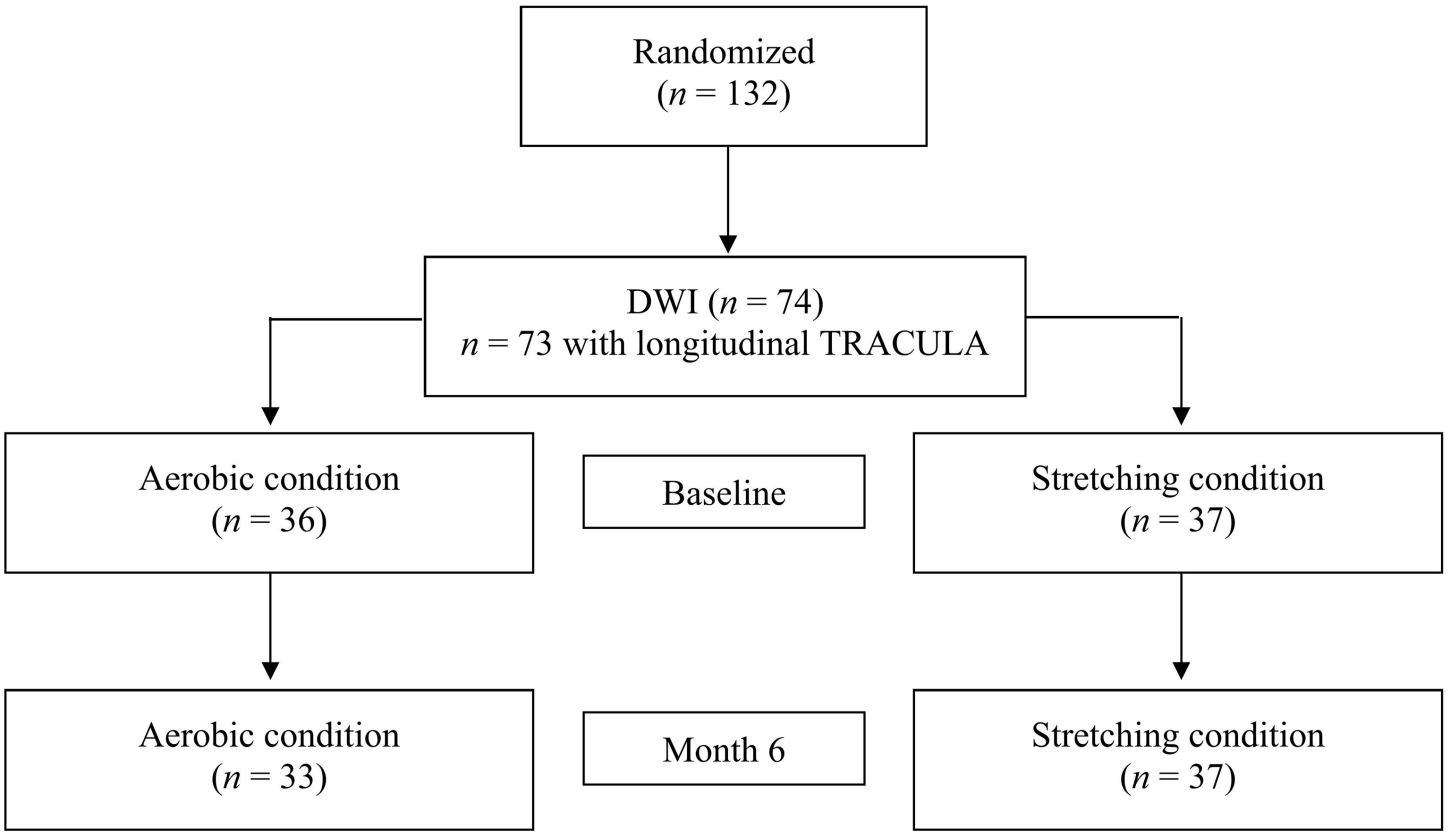
Flowchart.

Baseline characteristics of the participants with valid DWI data at baseline and post-intervention are summarized in Table 1 (see Figure 1 for the flowchart). Of the 74 participants with DWI data at baseline, 73 participants had valid data from the TRACULA longitudinal stream (AE group: *n* = 36, Stretching group: *n* = 37). At baseline, the group with valid DWI data did not differ from the DWI excluded group and the original study sample (*n* = 132) in terms of age, gender, education, IQ, or CRF. Also, at baseline, no statistically significant difference between intervention groups was found for sex, age, years of education, CRF, and the six cognitive domains assessed. Statistically significant differences between age group were found for variables known to decline with age such as CRF (*z* = −3.97, *p* < 0.001) (Hawkins and Wiswell, 2003), processing speed (*z* = −3.58, *p* = 0.001), and EF (*z* = −4.04, *p* = 0.001) (Kennedy and Raz, 2009). Of those who completed the intervention, eight participants (three in the AE group and five in the stretching/toning group) completed all 96 sessions; 65% of the AE group and 59% of the stretching/toning group completed more than 75% of the session. In terms of drop rate, only three participants (8%) dropped out in the AE group. Across the whole RCT, there were only three adverse events (1 = bruising from the phlebotomy, 2 = knee injuries).

**Table 1:** Baseline characteristics of the participants with longitudinal TRACULA data.

**Variables**	**All**	**Aerobic**	**Stretching**
*n* (% female)	70 (70)	33 (73)	37 (68)
Age	41.50 ± 14.62	43.94 ± 13.44	39.32 ± 15.44
Years of education	16.03 ± 2.46	15.36 ± 2.71	16.62 ± 2.07
VO_2_ max (ml/kg/min)	28.46 ± 6.54	27.59 ± 6.53	29.27 ± 6.54
Processing speed	0.05 ± 0.75	−0.01 ± 0.80	−0.09 ± 0.71
Episodic memory	0.08 ± 0.83	0.03 ± 0.88	0.13 ± 0.78
Working memory	0.12 ± 0.70	0.24 ± 0.60	0.23 ± 0.77
Language	0.02 ± 0.84	0.14 ± 0.80	−0.08 ± 0.88
Attention	−0.04 ± 1.01	0.08 ± 0.86	−0.14 ± 1.14
Executive function	−0.01 ± 0.81	−0.17 ± 0.99	0.15 ± 0.59

*Values are n (%) or mean ± SD. TRACULA, TRActs Constrained by UnderLying Anatomy*.

### Intervention Effect: Cognitive Performance and Cardiorespiratory Fitness

There were no significant Time × Group interaction or three-way interactions (Time × Intervention group × Age group or Time × Intervention group × Sex group) for any of the cognitive domains. Our analysis revealed a significant Time × Group interaction [*F*_(1,59)_ = 5.21, *p* = 0.026, $\eta_{\text{p}}^{2}$ = 0.081] for the CRF measure. The AE group (CRF_T1_ = 27.59 ± 6.53, CRF_T2_ = 30.36 ± 7.01) showed a statistically significant increase in the CRF value, in comparison with the stretching group (mean CRF _T1_ = 29.27 ± 6.54, mean CRF _T2_ = 30.00 ± 8.46). There were no significant three-way interactions (Time × Intervention group × Age group or Time × Intervention group × Sex group) for the CRF value.

### Intervention Effect: White Matter Tract Microstructural Measures

**Figure 2:**
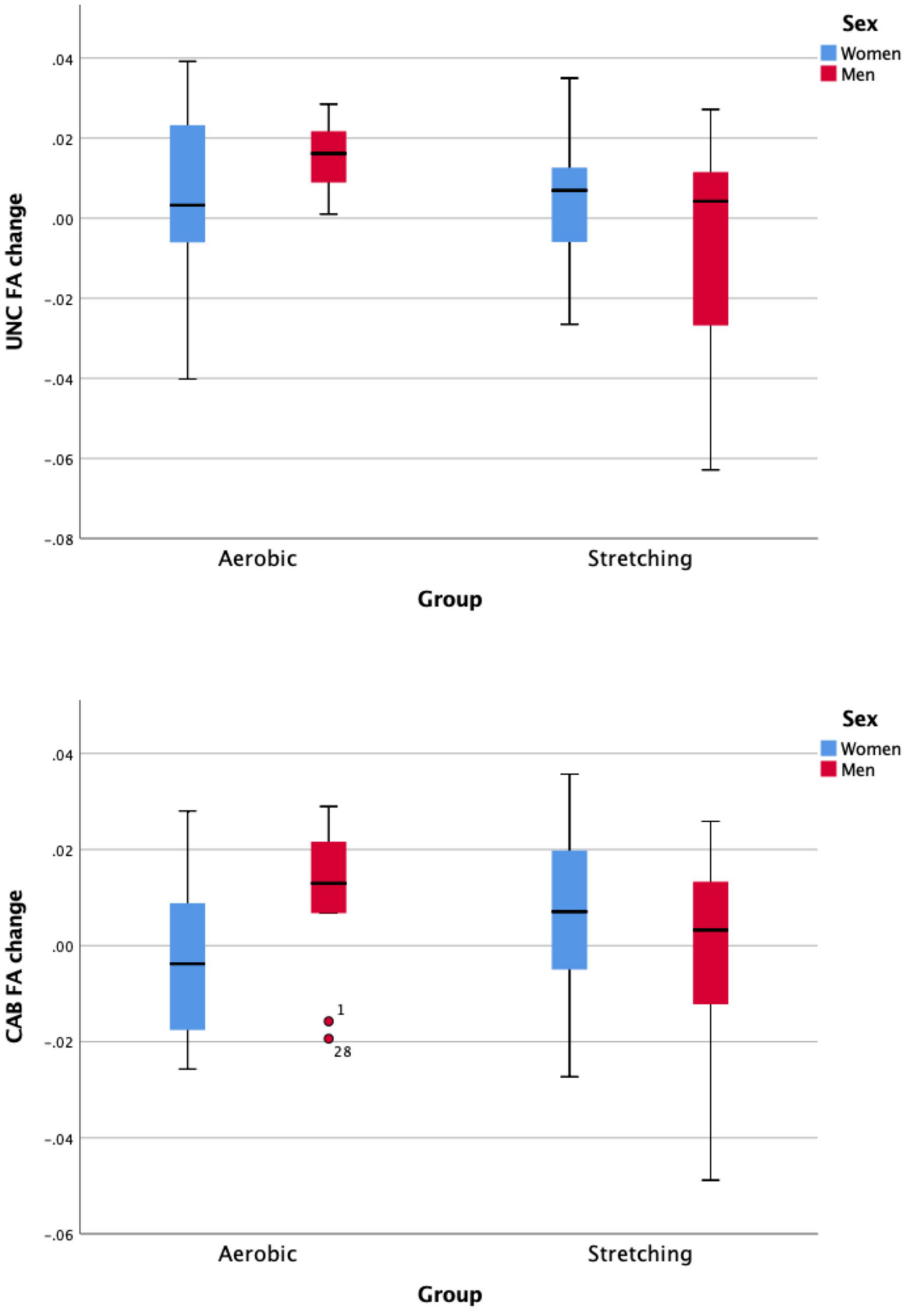
Sex-difference of the intervention effect in terms of change on the white matter tract fractional anisotropy of the uncinate fasciculus (UNC) and the Cingulum—angular (infracallosal) and bundle (CAB).

The intervention effect on the WMM measures (FA and MD) is summarized in Table 2. While non-significant after correcting for multiple comparisons, a significant (based on a *p* < 0.05) two-way interaction (Time × Intervention group) was found for the UNC FA [*F*_(1,58)_ = 6.23, *p* =0.015, $\eta_{\text{p}}^{2}$ =0.097, corrected *p* = 0.16]. Follow-up univariate analyses revealed that FA increases significantly in the UNC for the AE group [*F*_(1,32)_ = 8.23, *p* = 0.007, $\eta_{\text{p}}^{2}$ = 0.205], but not in the stretching group [*F*_(1,36)_ = 0.39, *p* =0.84, $\eta_{\text{p}}^{2}$ = 0.001]. There was no significant three-way interaction (Time × Intervention group × Age group) for any of the tract FA. While non-significant after correcting for multiple comparisons, a significant (based on a *p* < 0.05) three-way interaction (Time × Intervention group × Sex group) for the FA in the CAB [*F*_(1,58)_ = 5.96, *p* = 0.018, $\eta_{\text{p}}^{2}$ = 0.093, corrected *p* = 0.19] and the UNC [*F*_(1,58)_ = 5.57, *p* = 0.02 $\eta_{\text{p}}^{2}$ = 0.088, corrected *p* = 0.12] was measured. Follow-up univariate analyses revealed that in the AE group, the respective mean change for the FA of the CAB and the UNC differs between sex, as men had higher FA post-intervention for both tracts (see Figure 2).

**Table 2:** Intervention effect on the white matter tract microstructural in terms of mean percentage change.

	**FA**				**MD**			
	**Aerobic Δ**	**Stretching Δ**	***F***	***p***	**Aerobic Δ**	**Stretching Δ**	***F***	***p***
CST	0.82	0.35	1.16	0.29	−0.68	0.63	5.64	0.021
ILF	0.57	−0.32	0.74	0.39	−0.34	0.27	1.52	0.22
UNC	2.35	0.26	6.23	0.015	−0.80	0.38	5.23	0.026
ATR	0.30	0.20	0.19	0.67	−0.35	0.43	5.56	0.022
CCG	−0.09	1.37	2.23	0.14	−0.31	−0.38	0.00	1.00
CAB	0.07	1.14	0.21	0.64	−0.31	−0.07	0.56	0.46
SLFP	0.14	0.60	1.53	0.22	−0.51	−0.22	0.23	0.63
SLFT	0.47	0.76	0.13	0.72	−0.75	−0.09	1.22	0.27
FMAJ	0.46	0.61	0.08	0.77	−1.20	−0.16	1.29	0.26
FMIN	0.92	0.94	0.44	0.50	−1.15	−0.55	1.26	0.27
FX	−0.12	−0.66	0.24	0.63	−0.46	0.86	4.80	0.03

*Δ, mean percentage change; CST, corticospinal tract; ILF, inferior longitudinal fasciculus; UNC, uncinate fasciculus; ATR, anterior thalamic radiation; CCG, cingulum–cingulate gyrus (supracallosal) bundle; CAB, cingulum–angular (infracallosal) bundle; SLFP, superior longitudinal fasciculus–parietal bundle; SLFT, superior longitudinal fasciculus–temporal bundle; FMAJ, forceps major; FMIN, forceps minor; FX, fornix*.

While non-significant after correcting for multiple comparisons, significant (based on a *p* < 0.05) two-way interaction (Time × Intervention group) was found for the ATR MD [*F*_(1,58)_ = 5.56, *p* = 0.022, $\eta_{\text{p}}^{2}$ = 0.088], the CST MD [*F*_(1,58)_ = 5.64, *p* = 0.021, $\eta_{\text{p}}^{2}$ = 0.089], the UNC MD [*F*_(1,58)_ = 5.23, *p* = 0.026, $\eta_{\text{p}}^{2}$ = 0.083], and the fornix MD [*F*_(1,58)_ = 4.80, *p* = 0.032, $\eta_{\text{p}}^{2}$ = 0.077]. Follow-up univariate analyses revealed that the MD of the UNC decreased significantly post-intervention for the AE group [*F*_(1,32)_ = 5.78, *p* = 0.022, $\eta_{\text{p}}^{2}$ = 0.153], but not in the stretching group [*F*_(1,36)_ = 0.63, *p* = 0.43, $\eta_{\text{p}}^{2}$ = 0.017]. There were no significant three-way interactions (Time × Intervention group × Age group or Time × Intervention group × Sex group) for any of the tract MD.

### Correlation Analysis Between Measures of Change

As no significant change was observed in terms of cognitive performance, only the correlations between the intervention-related percentage change in terms of WMM and CRF were computed. For the AE group, correlation analysis revealed a significant positive correlation between CRF and the SLFT FA change, *r* = 0.41, *p* = 0.023, and a significant negative correlation between CRF and the SLFT MD change, *r* = −0.37, *p* = 0.04. However, these correlations did not survive correction for multiple comparisons. For the stretching group, no significant correlation was found.

## Discussion

The goal of the present study was to evaluate the impact of an AE program on the WMM in a sample of young and middle-aged adults, using a subset (participants who have DWI) of an RCT (Stern et al., 2019b). No significant group-level change in WM tract microstructure following an AE training was observed. No significant correlation was found between intervention-related percentage change in terms of WMM, CRF, and cognitive performance.

The lack of group-level change in WM tract microstructure following an AE training replicates the finding reported by Voss et al. (2013) and Clark et al. (2019) in healthy older adults and those by Tarumi et al. (2020) using TRACULA with a sample of older adults with amnestic mild cognitive impairment. In this RCT, a lack of WMM improvement was also observed when analyzing the results by age group or biological sex. The lack of an age group effect might be related to how the age group was split. As our sample is predominantly composed of women, future studies examining the effect of biological sex should target an equivalent number of men and women and use sex-disaggregated analyses. No significant correlation was found between change in cognitive performance and change in WMM. Contrary to the finding in older adults by Voss et al. (2013), change in CRF in the AE group did not correlate with the respective FA and MD change.

While not significant after controlling for multiple comparisons, an increase in FA and a decrease in MD of the uncinate fasciculus were observed post-intervention in the AE group in comparison with the stretching group. This null effect after correction for multiple corrections is unlikely to be attributable to any reliability issue in the TRACULA approach: FA for the 18 major WM tracts from TRACULA has been shown to exhibit greater reliability in the longitudinal processing technique, as used in this study, than using a cross-sectional technique (Yendiki et al., 2016), with an error of 5% for longitudinal vs. 11% for cross-sectional approach.

UNC WMM change also did not correlate with change in cognitive performance or CRF after correction for multiple comparison. While the UNC WMM has been associated with cardiovascular health in healthy adults (Cox et al., 2019; Fuhrmann et al., 2019) (but see Tian et al., 2014 for a null effect), future studies should investigate other mechanisms by which UNC WMM could be improved by an AE training. Interestingly, interventions that do not target CRF have previously reported change in the UNC FA. For example, a pilot study evaluating the effect of a mindfulness intervention in healthy adults aged 18–65 (Holzel et al., 2016) has reported an increase FA in the right UNC (as computed by the TRACULA tool), and a 3-month cognitive training has shown increased FA in the left UNC (Chapman et al., 2015).

Limitations of this study include common limitations related to DTI measures (Mori and Zhang, 2006) and a relatively small sample size (particularly for the AE group). Furthermore, the homogeneity of the sample in terms of biological sex and fitness level could have reduced the generalizability of our finding. Lastly, other sources of influences on the differences in change in diffusivity measurement between intervention groups were not measured. For example, different states of hydration have been shown to affect WM (Streitburger et al., 2012), and the participation to the AE group could have induced a change in the state of hydration of the participant (Trangmar and Gonzalez-Alonso, 2019). A strength of the present study includes the use of an objective assessment of CRF based on GXT, the use of an extensive neuropsychology battery, the presence of a randomization process, the use of supervised training, the presence of an active control group, and the selection of an understudied sample of healthy middle-aged adults.

The present results of this study are in accordance with similar studies that did not show evidence for the possible benefit of participating in an AE program on WMM in healthy adults. Future studies need larger sample sizes to address issues such as low statistical power, particularly when biological sex is taken into account. Use of more sensitive techniques to assess WMM is also encouraged. Participants presenting modifiable risk factors that affects WMM integrity (Wassenaar et al., 2019), such as hypertension, obesity, diabetes, and smoking, could potentially benefit from physical exercise or a combination of activities. Lastly, to better understand the specific contribution of CRF change to possible WMM and cognitive changes, results of similar RCT could categorize participants in the AE training as “responders or non-responders” based on their CRF change post-intervention.

## Data Availability Statement

The data from this randomized clinical trial, including anonymized participant-level and study-level data (analyzable data sets) and other information (such as protocols), will be shared with qualified researchers as necessary for conducting legitimate research through direct request from the study principal investigators (YS at ys11@columbia.edu or RPS at rps7@cumc.columbia.edu).

## Ethics Statement

The study was reviewed and approved by the Institutional Review Board of New York State Psychiatric Institute. The participants provided their written informed consent to participate in this study.

## Author Contributions

YS and RPS: conceptualization and funding acquisition. DP, YG, and PL: data curation. DP, YG, and SL: formal analysis. DP, YS, and YG: writing—original draft preparation. All authors contributed to the article and approved the submitted version.

## Conflict of Interest

The authors declare that the research was conducted in the absence of any commercial or financial relationships that could be construed as a potential conflict of interest.
